# Decomposing biophotovoltaic current density profiles using the Hilbert–Huang transform reveals influences of circadian clock on cyanobacteria exoelectrogenesis

**DOI:** 10.1038/s41598-022-15111-y

**Published:** 2022-06-29

**Authors:** Tonny Okedi, Kamran Yunus, Adrian Fisher

**Affiliations:** 1 Department of Chemical Engineering and Biotechnology, University of Cambridge, Phillipa Fawcett Drive, Cambridge, CB3 0AS UK; 2 Cambridge Center for Advanced Research and Education in Singapore (CARES), 1 Create Way, #05-05 CREATE Tower, Singapore, 138602 Singapore

**Keywords:** Circadian rhythms, Photosynthesis, Fuel cells, Energy science and technology

## Abstract

Electrons from cyanobacteria photosynthetic and respiratory systems are implicated in current generated in biophotovoltaic (BPV) devices. However, the pathway that electrons follow to electrodes remains largely unknown, limiting progress of applied research. Here we use Hilbert–Huang Transforms to decompose *Synechococcus elongatus* sp. PCC7942 BPV current density profiles into physically meaningful oscillatory components, and compute their instantaneous frequencies. We develop hypotheses for the genesis of the oscillations via repeat experiments with iron-depleted and 20% CO${_2}$ enriched biofilms. The oscillations exhibit rhythms that are consistent with the state of the art cyanobacteria circadian model, and putative exoelectrogenic pathways. In particular, we observe oscillations consistent with: rhythmic D1:1 (photosystem II core) expression; circadian-controlled glycogen accumulation; circadian phase shifts under modified intracellular %ATP; and circadian period shortening in the absence of the iron-sulphur protein LdpA. We suggest that the extracted oscillations may be used to reverse-identify proteins and/or metabolites responsible for cyanobacteria exoelectrogenesis.

## Introduction

There is a critical need for CO${_2}$ capture and abatement technologies in order to meet global climate change goals for less than 2 $^{\circ }$C of warming. Biophotovoltaic systems (BPVs) which employ microorganisms that perform oxygenic photosynthesis have emerged as one such potential technology. A BPV is an electrochemical cell in which at least one electrode is catalysed by photosynthetic microorganisms such as algae and cyanobacteria, that absorb CO${_2}$ from the atmosphere, reduce it into carbon products during photosynthesis, and store it as biomass. Electrons generated from the photosynthetic process have been implicated in the enhanced current generated in these devices under illumination (photocurrent), and in the basal current in the dark (dark current) from oxidation of the stored biomass e.g., via respiration or the oxidative pentose phosphate pathway^1–4^.

**Figure 1:**
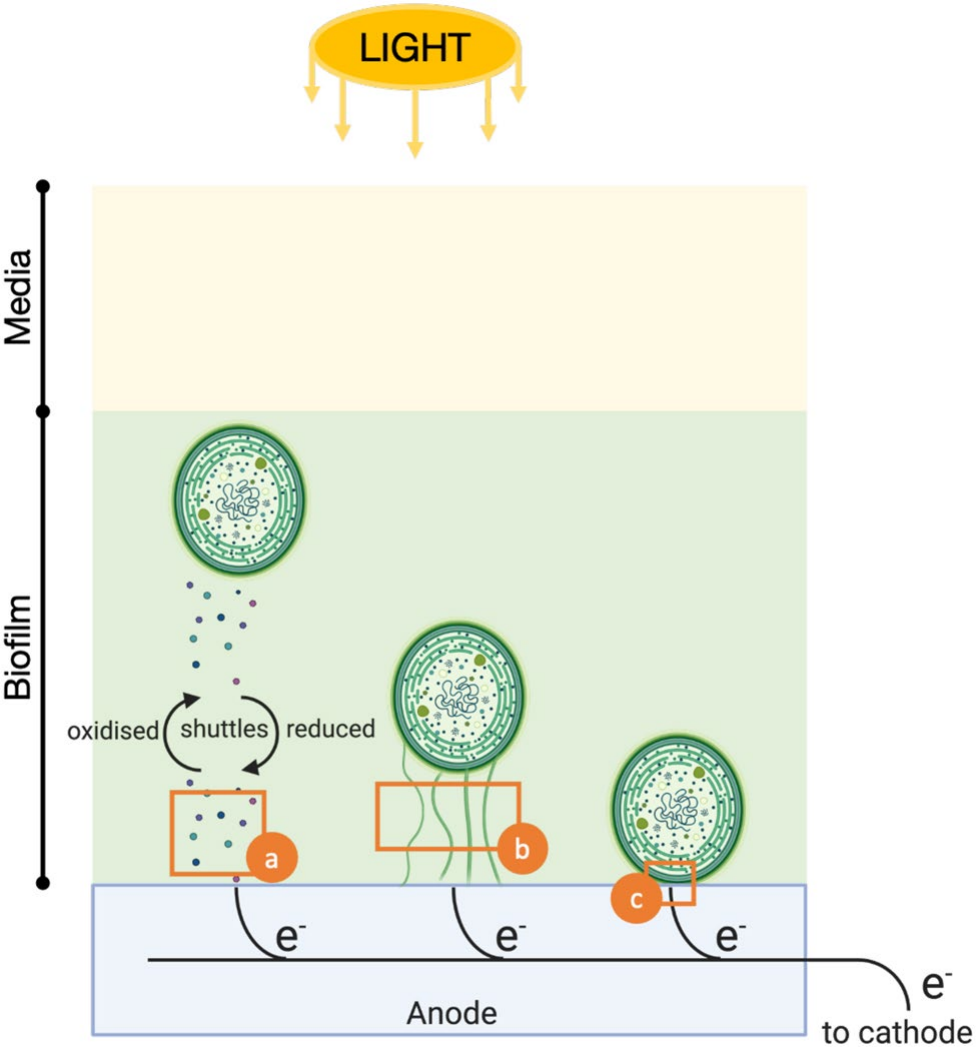
Putative terminal electron transport step in cyanobacteria. (**a**) indirect electron transport via endogenous electron mediators excreted by the cells; (**b**) direct electron transfer via extracellular appendages that traverse the cell cytoplasmic membrane and outer cell wall in contact with the electrode; and (**c**) direct electron transfer via redox active proteins embedded in the cell outer membrane. Schematic is not to scale.

Key to delivering BPVs is understanding why the microorganisms donate electrons to their surroundings (exoelectrogenesis) and the complex electron flows from the photosynthetic and respiratory electron transport chains, to the non-living electrodes (Fig. 1c). This remains a major hurdle in advancing the fundamental understanding needed to develop more efficient light conversion, and to enable applied research to realise the technology at a commercial scale.

To date, electron flows have been primarily investigated via spectroscopy and electrochemical techniques such as chronoamperometry and cyclic voltammetry studies on mutant, stressed or inhibited cells^2–8^. Computational models and tools are being developed that can be used to help interpret experimental data and run rapid simulations towards understanding the electron flows^9–11^. However, due to knowledge gaps, the non-linear and nonstationary nature of bioelectrochemical processes, and difficulties in the in-situ measurement of key variables, it has proved difficult to develop robust first-principle computational models. To overcome these challenges, the authors recently applied deep learning, in particular Long Short-Term Memory (LSTM) networks, to predict the one-step-ahead seasonal current density in BPVs using a three step process: (1) decomposing the observed current density into a trend, seasonal and irregular component via Seasonal and Trend Decomposition using Locally Estimated Scatterplot Smoothing or LOESS (STL); (2) training the LSTM network with the seasonal component; and (3) predicting the one-step-ahead seasonal current density using the trained network^12^.

While satisfactory results were achieved, the LSTM model was limited to predicting the repeatable seasonal current density. In order to forecast the observed current density, a separate model for the erratic and difficult-to-predict irregular component is required. The LSTM forecasts were shown to be unsatisfactory without the removal of the irregular component^12^. Furthermore, the physical meanings of the trend, seasonal and, particularly, irregular components obtained via STL are unclear. It is therefore imperative to find an alternative decomposition approach that results in more regular, physically meaningful sub-components in order to develop more robust models via LSTMs or other time series modelling techniques. To achieve this, the authors previously proposed Empirical Mode Decomposition (EMD), a time series decomposition method applied during the Hilbert–Huang transform (HHT), an energy-time-frequency analysis technique^12^.

The frequency domain can reveal characteristics of a signal that cannot be distinguished by time-domain analysis (such as STL) only. The Fourier transform is the most well known and commonly used frequency-domain analysis technique due to its simplicity^13,14^. The fundamental idea behind the Fourier transform is that signals, *X*(*t*), can be represented as the linear superposition of trigonometric functions (e.g., sine and cosine) of discrete frequencies ($\omega$). Thus, the requirements of the Fourier analysis is that the signal is periodic or stationary data (i.e., constant, unchanging mean), and a linear system (i.e., follows the principle of superposition)^13^. This is often not the case for bioelectrochemical data since electrochemical and biological processes are both highly nonstationary and/or nonlinear^14,15^. To overcome this challenge, the short-time Fourier transform (STFT) was developed. STFT consists of applying the Fourier transform using a fixed sliding window along the time series to locate frequency events on the time axis^13^. However, it must then be assumed the data is piece-wise stationary over the span of the window. It is difficult to guarantee that the window size applied always results in a stationary section of the data. Furthermore, the STFT is subject to the Heisenberg uncertainty principle. There is a trade-off between time resolution (good time resolution requires short time windows) and frequency resolution (good frequency resolution, 1/T, requires long time windows)^13,14^. If window size is too long, then ability to localise frequency characteristics is lost. If window size is shorter than the longest frequency oscillations, then these may not be detected. Wavelet analysis overcomes some of the time-frequency trade-offs of the STFT. It can be conceptualised as an adjustable window STFT^13,14^. Synchrosqueezing (SST) builds on Wavelet analysis by improving the selection of appropriate scales for the analysis^16,17^. Unimportant wavelets (in time and scale) are removed using thresholds. Similarly, the Empirical Wavelet Transform is an adaptive wavelet technique aimed at improving Wavelet analysis^17^. Wavelet approaches however have the disadvantage of hard band-limits and in some cases, unknown wavelets^17^. Other Fourier-derived techniques include the Fourier Decomposition Method (FMD) and the Variation Mode Decomposition for which the interested reader is referred to the cited literature^17,18^.

The Hilbert–Huang transform is a non-Fourier two-step time series analysis technique for non-linear and/or nonstationary signals such as exoelectrogenic currents^13^. The first step is a pre-processing EMD stage. The goal of EMD is to expand a nonstationary signal made up of multiple, superimposed, dynamic oscillations, into a finite set of sub-components or intrinsic mode functions (IMFs). Each IMF (or simply mode) includes oscillations of a characteristic, physically meaningful, timescale (meaning the time between oscillatory peaks)^13^. This allows the Hilbert transform to be applied to each IMF to obtain meaningful instantaneous frequencies along its time course. Thus, HHT is an amplitude-frequency-time technique that allows the identification of events on both the time and frequency axes.EMD has heuristic elements that make it difficult to analyse mathematically. For example, EMD-based algorithms are sensitivity to the methods used to (i) obtain the extrema and (ii) interpolate the upper and lower envelopes, as well as (iii) the sifting stopping criteria (see “Methods”). Despite these disadvantages, HHT is simple to use and has been applied widely within the biological community. HHT has been used to process electrocardiograms (ECG) and electroencephalographs (EEG) in medicine including the study of circadian rhythms in humans^19,20^. In the field of bioelectrocatalysis, HHT has been applied to analyse the current generated in bioelectrochemical single molecule sensors to obtain valuable information on the covalent and non-covalent interactions between the molecule and the nanopore^14,21–23^.

It was hypothesised that by applying the HHT to current density profiles from unmediated BPVs, distinct oscillatory patterns can be isolated, from which characteristic timescales and frequencies intrinsic to natural exoelectrogenesis may be identified. In this work, the decomposition step is implemented with a modified EMD algorithm called Improved Complete Ensemble EMD with Adaptive Noise (ICEEMDAN), developed to result in more physically meaningful IMFs (see “Methods” for more details)^19^. To help decode the physical meaning of the extracted IMFs, BPVs were operated in three different conditions known to affect exoelectrogenesis in distinct ways: (1) standard laboratory growth conditions (control); (2) iron depleted media; and (3) iron depleted media in the presence of 20% CO${_2}$. BPVs operated with iron-depleted *Synechococcus elongatus* sp. PCC7942 (*S. elongatus* henceforth) biofilms exhibit significantly larger currents but a markedly reduced light response^7,24^. Peculiarly, BPVs operated with iron-depleted cultures in the presence of 20% v/v CO${_2}$ and with ferricyanide as an exogenous mediator, exhibit higher current output in the dark than in the light^24^. The enhanced currents under iron starvation was suggested as evidence of the over-expression of a redox-active protein involved in iron acquisition at the outer membrane level^7^. The enhanced dark current in the presence of 20% CO${_2}$ was suggested to be evidence of preferential utilisation of stored energy for exoelectrogenesis.

Throughout the paper, Fe(+)|Air refers to the control condition (black lines in graphs), Fe(−)|Air refers to iron depleted cultures/biofilms in atmospheric air (green lines in graphs), and Fe(−)|20% CO${_2}$ refers to iron depleted cultures/biofilms in the presence of 20% CO${_2}$ (blue lines in graphs). Three replicate cultures (different independent samples prepared in the same way) were grown for each growth condition, and all measurements (including electrochemical measurements) were performed on each independent culture; for each condition, reported results are arithmetic means across the three replicates. The additional suffix or subscript |3 h refers to operation under a 3 h:3 h light-dark period, while |12 h refers to a operation under a 12 h:12 h (diel) light-dark period. Finally, borrowing from terminology used in chronobiology,* dusk* refers to light-to-dark transitions,* dawn* to dark-to-light transitions,* day *to the illuminated interval of a period, and *night *the dark interval.

## Results

### Current density profiles

**Figure 2:**
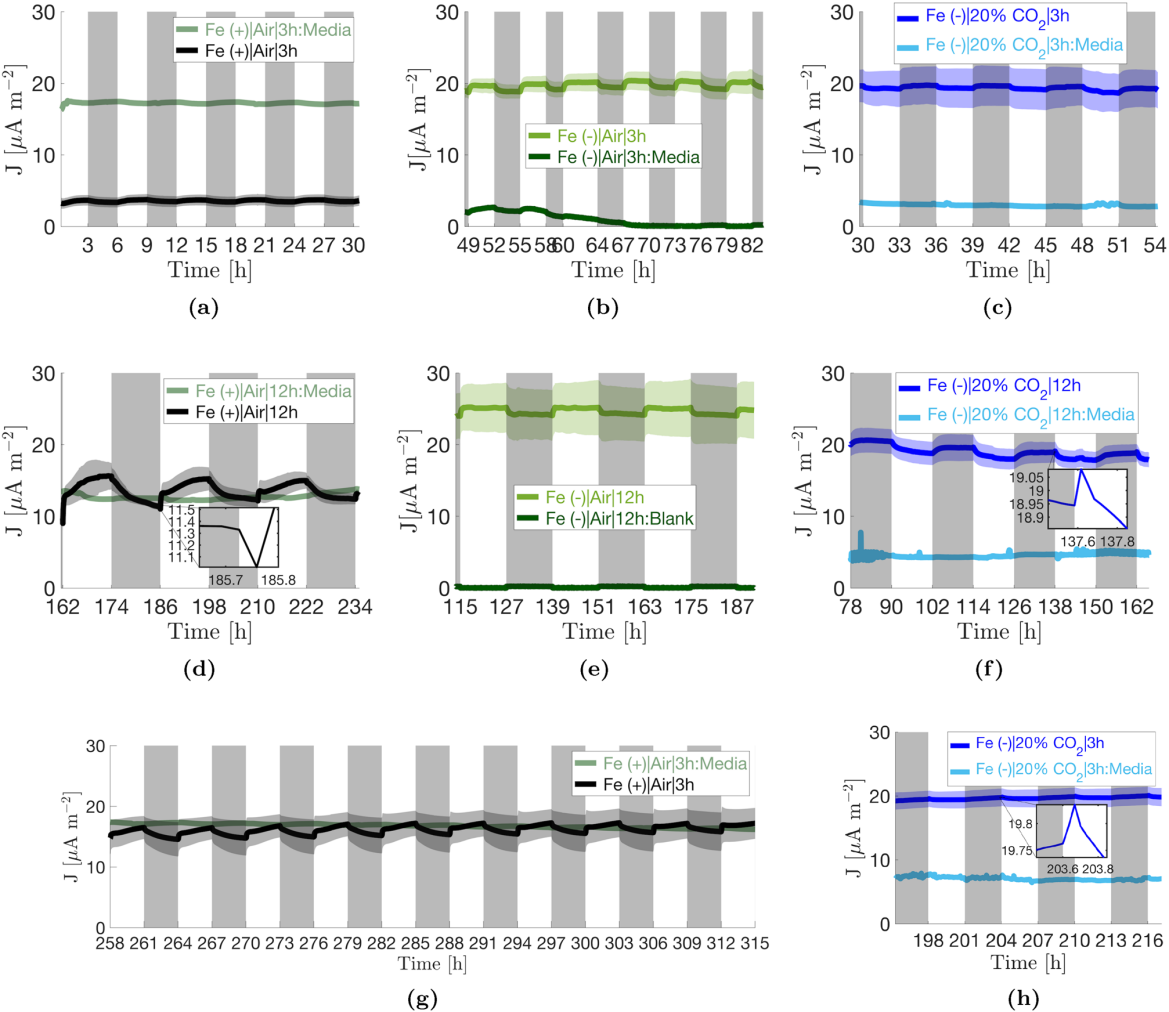
Current density profiles. Each profile shows the mean of three independent replicates $\pm 1$ standard error of the mean (shaded areas). Current profiles of abiotic BPVs inoculated with the corresponding fresh media are also shown. BPVs were operated with the procedure shown in Table 2. Profiles (**a**)–(**c**) and (**g**)–(**h**) show operation under 3 h:3 h light-dark periods while (**d**)–(**f**) show operation under 12 h:12 h light-dark periods. Profiles (**g**)–(**h**) were recorded after replenishment of respective media into the BPVs. The time gaps between the corresponding 3 h:3 h profiles and the 12 h:12 h profiles includes $\approx$ 10 h during which polarisation curves where measured (Supplementary Fig. S10), and the remaining time the duration of voltage recovery and stabilisation following reconnection of the 33 M$\Omega$ external resistors. This duration varied by operating condition. In (**b**), a light timer error resulted in a two- rather than three-hour dark interval at 58 h, and consequently a four- rather than three-hour light interval ending at 64 h.

Figure 2 shows the measured current density profiles for the three conditions investigated following the operating procedure in Table 2 (“Methods”). Current densities from abiotic BPVs inoculated with the corresponding fresh medium for each condition are also shown to give an indication of the background current. It should be noted that the magnitudes of the backgrounds are exaggerated because in the biotic devices, the cells assimilate redox active species in the medium over time, reducing their concentration. This is particularly true for the Fe(+)|Air condition, where redox active ferric ions are assimilated by the cells. We previously estimated that the concentration of ferric ions in fresh BG11 medium reduces by half within four days of culturing and reduces to almost zero within 11 days under similar growth conditions^25^. Similar rates of ferric ion depletion rates in *S. elongatus* cultures have been independently reported from atomic absorption spectroscopy concentration measurements^26^.

#### Non-stressed cells exhibit a two-sloped exoelectrogenic signal after a ca. 40 h time lag

The Fe(+)|Air BPVs did not produce significant current during the first 30 h of operation under load (Fig. 2a). After measurement of a polarisation curve in the dark (Supplementary Fig. S10a) and reconnection of the 33 $M\Omega$ external resistor, the current densities increased erratically from around 40 h and began to stabilise at around 140 h (data not shown). Repeatable oscillations were observed in all three replicates after approximately 162 h, Fig. 2d. The time scales of the different current generation phases (negligible, erratic, stable) mirror the evolution of Fe(+)|Air culture growth from lag to stationary phase (Supplementary Fig. S2). It is therefore hypothesised that: (i) the negligible current density in the first 30 h is due to cells in the lag phase prioritising accumulation of resources, including reducing power, for cell proliferation; (ii) the erratic increase in current density is due to biofilms in exponential phase, where there is rapid growth in electrochemically active biomass in the biofilm; and (iii) the stable currents are achieved when the biofilm reaches stationary phase where concentration of active biomass in the biofilm remains constant, and gradual changes in cellular morphology contribute to changes in the mean current level by influencing electron export rates^25^. Patterns previously reported when operating under a diel (24 h) light-dark period were observed, namely a dip and recovery in current density at dawn (inset, Fig. 2d), and a period-to-period decay in the magnitude of the photocurrent^12^. Following the dip, photoresponse was two-sloped, with an initial steep spike in current, followed by a more gradual increase over the remaining duration of the illuminated interval. In addition, the slope of the initial spike decayed substantially with each period, dropping from around 20 $\upmu$A $m^{-2}$ $h^{-1}$ at the 162 h dawn to around 6.5 $\upmu$ $m^{-2}$ $h^{-1}$ at the 234 h dawn (Supplementary Fig. S11d).

To test whether the decaying photoresponse in the Fe(+)|Air BPVs was due to the depletion of the Fe(+)|Air medium, BPVs were replenished with fresh BG11 media, reconnected to 33 M$\Omega$ resistors and operated with a 3 h:3 h light-dark period (Fig. 2g). The mean current density maintained its level, and even increased slowly over time. The slope of the initial positive photoresponse also showed significant recovery reaching approximately 13 $\upmu$A $m^{-2}$ $h^{-1}$ at the 295 h dawn (Supplementary Fig. S11g). However, the second, gradual photoresponse was not recovered, and overall photoresponse remained low. The dip in current at dawn was absent under the 3 h:3 h light-dark regime confirming previously reported results^12^.

#### Iron stress expedites activation of exoelectrogenesis, amplifies mean current output, but reduces light gradients

In line with previous results, Fe(−)|Air BPVs had higher current densities versus the control, but a markedly reduced photoresponse (Fig. 2b,e). Current densities increased erratically for the first 40–45 h upon connection to a 33 M$\Omega$ external resistor (data not shown). All 3 replicates produced stable and repeatable current oscillations from 49 h onwards. This was substantially quicker than in the control. Similar to the Fe(+)|Air devices, the evolution from erratic to stable current output mirrors the evolution of Fe (–)|Air culture growth i.e., a substantially shorter lag phase ($< 6$ h) and transition to decline within 45 h of inoculation (Supplementary Fig. S2 and Supplementary Table S2). This further corroborates the hypothesis that stable currents are achieved when biofilms reach the stationary phase. At 82 h, the light-dark period was switched from 3 h:3 h to 12 h:12 h. It took approximately 33 h to reestablish stable and repeatable oscillations which were achieved in all three replicates from 115 h onwards (Fig. 2e). This was substantially quicker than in the control. The mean current density also increased slightly during this time. Unlike in the Fe(+)|Air|12 h BPVs, there was no dip in current at dawn and photoresponse was mono-sloped. The photocurrent therefore plateaued after the initial rise. As in the control, the slope of the photoresponse decayed from period to period, albeit at a much slower rate. The slope was also less steep, reducing from approximately 4 $\mu$A $m^{-2}$ $h^{-1}$ at the 49 h dawn, to 2 $\upmu A$ $m^{-2}$ $h^{-1}$ at the 187 h dawn (Supplementary Fig. S11b,e).

#### The presence of 20% CO${_2}$ further accelerates the start of exoelectrogenesis and amplifies the dark current

Fe(−)|20% CO${_2}$ BPV current densities were similar in magnitude to those of the Fe(−)|Air condition (Fig. 2c,f). Distinctively, the current was marginally higher in the dark than in the light, with a conspicuous lack of the enhanced photocurrent typical in BPVs. The results confirm observations previously reported in the research group, here also observed in unmediated devices^24^. Counterintuitively, there was a positive dark response at dusk, with a diminishing slope of less than 1.8 $\upmu$A $m^{-2}$ $h^{-1}$ (Supplementary Fig. S11c,f). With every subsequent dusk, the dark response became less prominent. Oppositely, an ephemeral positive photoresponse at dawn appeared over time, first seen at 114 h and slowly increased in prominence with each new dawn, inset of Fig. 2f. The Fe(−)|20% CO${_2}$ BPVs achieved stable and repeatable oscillations within the first 30 h of connection to a 33 M$\Omega$ external resistor (Fig. 2e). Following the polarisation curve at 54 h, the BPVs reestablished stable and repeatable oscillations within 12–14 h of reconnecting the 33 M$\Omega$ external resistor, much faster than the other two conditions (Fig. 2f). This is consistent with the rapid evolution of Fe(−)|20% CO$_2$ cultures to stationary phase (Supplementary Fig. S2 and Supplementary Table S2).

To check the persistence of the ephemeral positive photoresponse that first appeared at the 114 h dawn, the Fe(−)|20% CO${_2}$ BPVs were operated for a further 21 h under a 3 h:3 h light-dark period after the light polarisation at 162 h (Fig. 2h). The persistence of the photoresponse was confirmed as clearly seen in the derivative of the current density profile (Supplementary Fig. S11h). Further, it was observed that in the long term, the current profile became flatter and flatter as the positive dark response continued to diminish.

### Hilbert–Huang transforms

**Figure 3:**
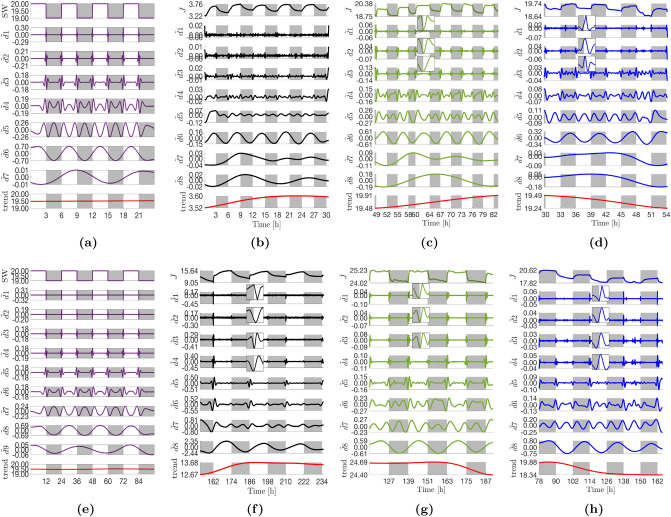
Intrinsic mode functions (IMFs) extracted via the ICEEMDAN algorithm. (**a**) SW|3 h. (**b**) Fe(+)|Air|3h. (**c**) Fe(−)|Air|3 h. (**d**) Fe(−)|20% CO${_2}$|3 h. (**e**) SW|12 h. (**f**) Fe(+)|Air|12 h. ((**g**) Fe(−)|Air|12 h. (**h**) Fe(−)|20% CO$_2$|12 h. For each decomposition, the top panel shows the mean current density profile, $\bar{J}$, as reported in Fig. 2, the central panels $\tilde{d}1-8$ show the extracted IMFs, and the bottom *trend* panel shows the final residue from the decomposition process *rK* (red line). Insets show zooms of the highest frequency oscillations at dawn for improved clarity. It should be noted that the widths and heights of the insets reflect varying durations (increasing as you move downward from $\tilde{d}1$) and amplitudes. Light changes are superimposed as the backgrounds of the panels (light on white and light off grey).

**Figure 4:**
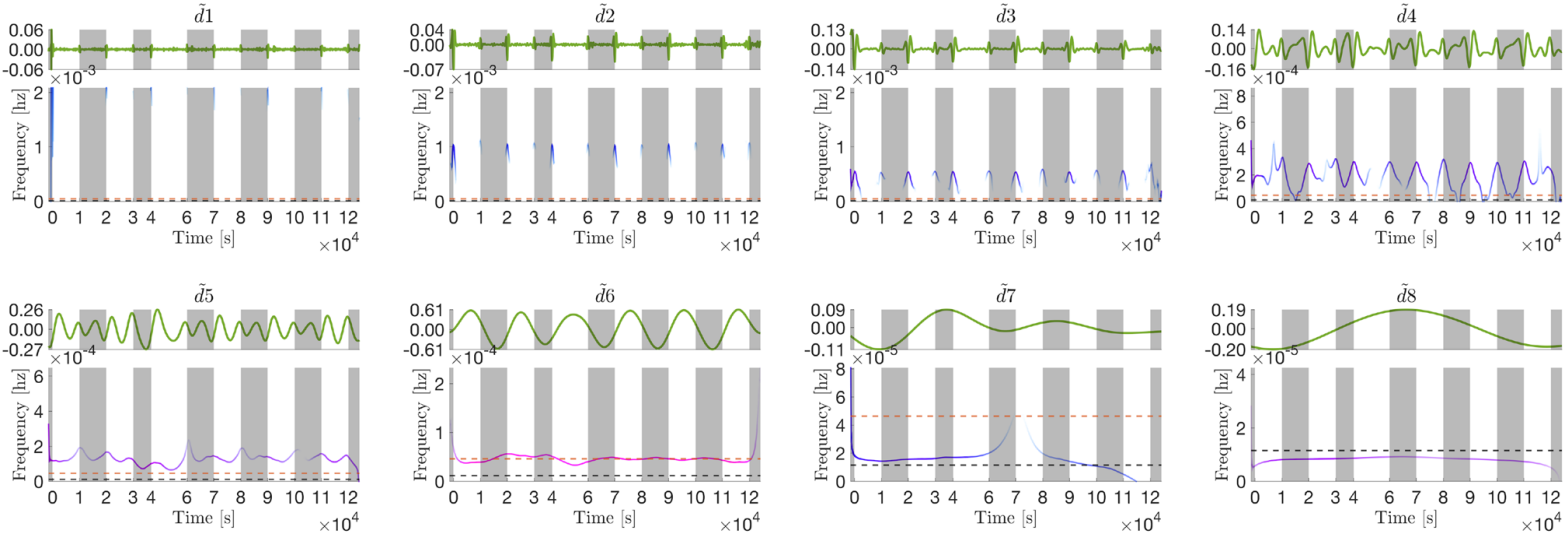
IMFs in Fig. 3c extracted from the Fe(−)|Air|3 h current density profile (top panes) and their corresponding instantaneous frequency profiles (bottom panes). The instantaneous frequency profiles are contoured with the instantaneous energy of the oscillations (see Fig. 5 for the colour bar). The orange dashed line marks the frequency of the light-dark period, while the black dashed line marks the diel (24 h) frequency. When plotted on one axis, the contoured frequency profiles above form the Hilbert spectrum displayed in Fig. 5c. Note the x-axes origins have been zeroed and time is shown in seconds (standard representation).

**Figure 5:**
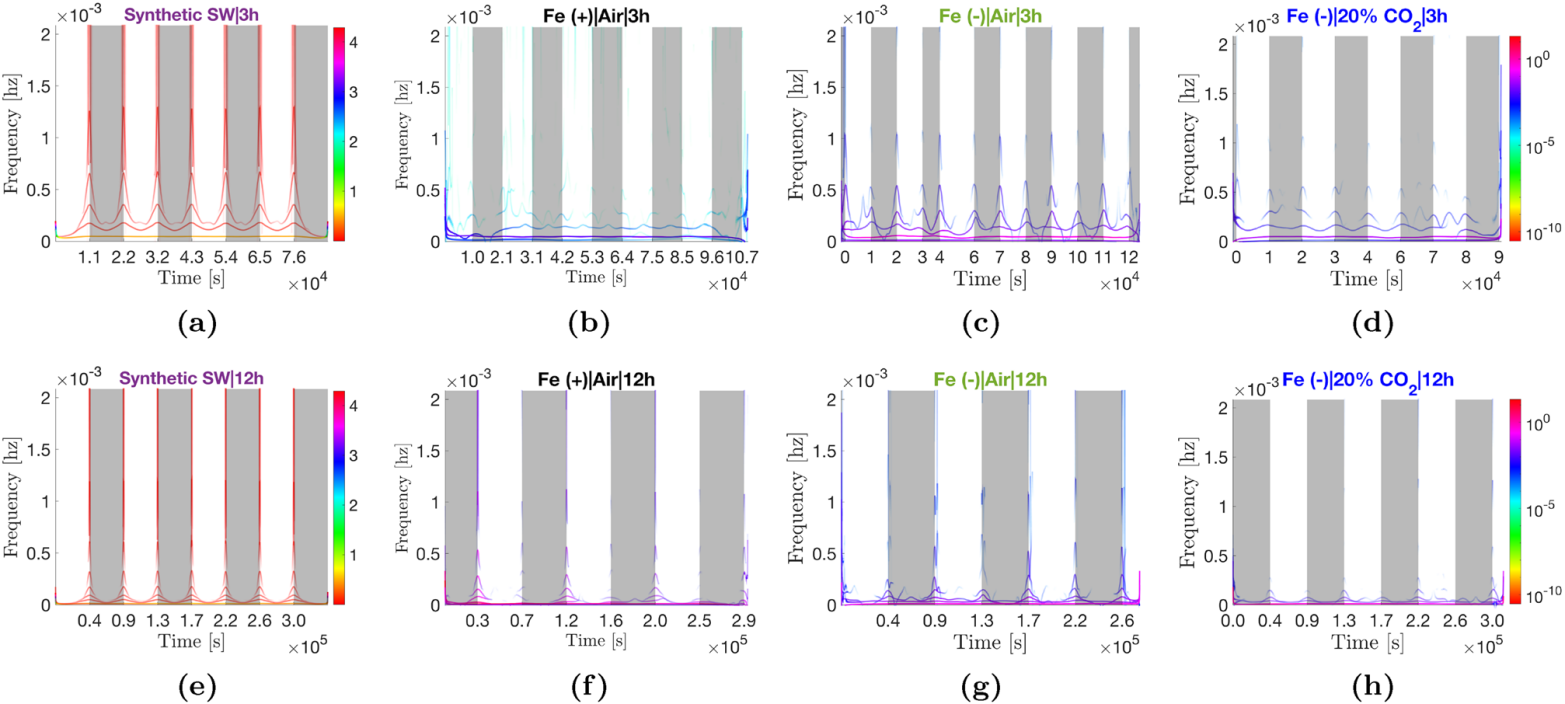
Hilbert spectra of the different current density profiles showing the energy-frequency-time distributions. Each spectrum is a single-axis plot of the full set of contoured instantaneous frequency profiles computed from the IMFs of the corresponding operating condition (Fig. 3). For example, (**c**) shows the single-axis plot of the instantaneous frequency profiles shown in Fig. 4. The y-axis is limited to the maximum frequency than can be resolved with a 2-min sampling interval (see Eq. 7). Note the x-axes origins have been zeroed and time is shown in seconds (standard representation of the Hilbert spectrum).

**Figure 6:**
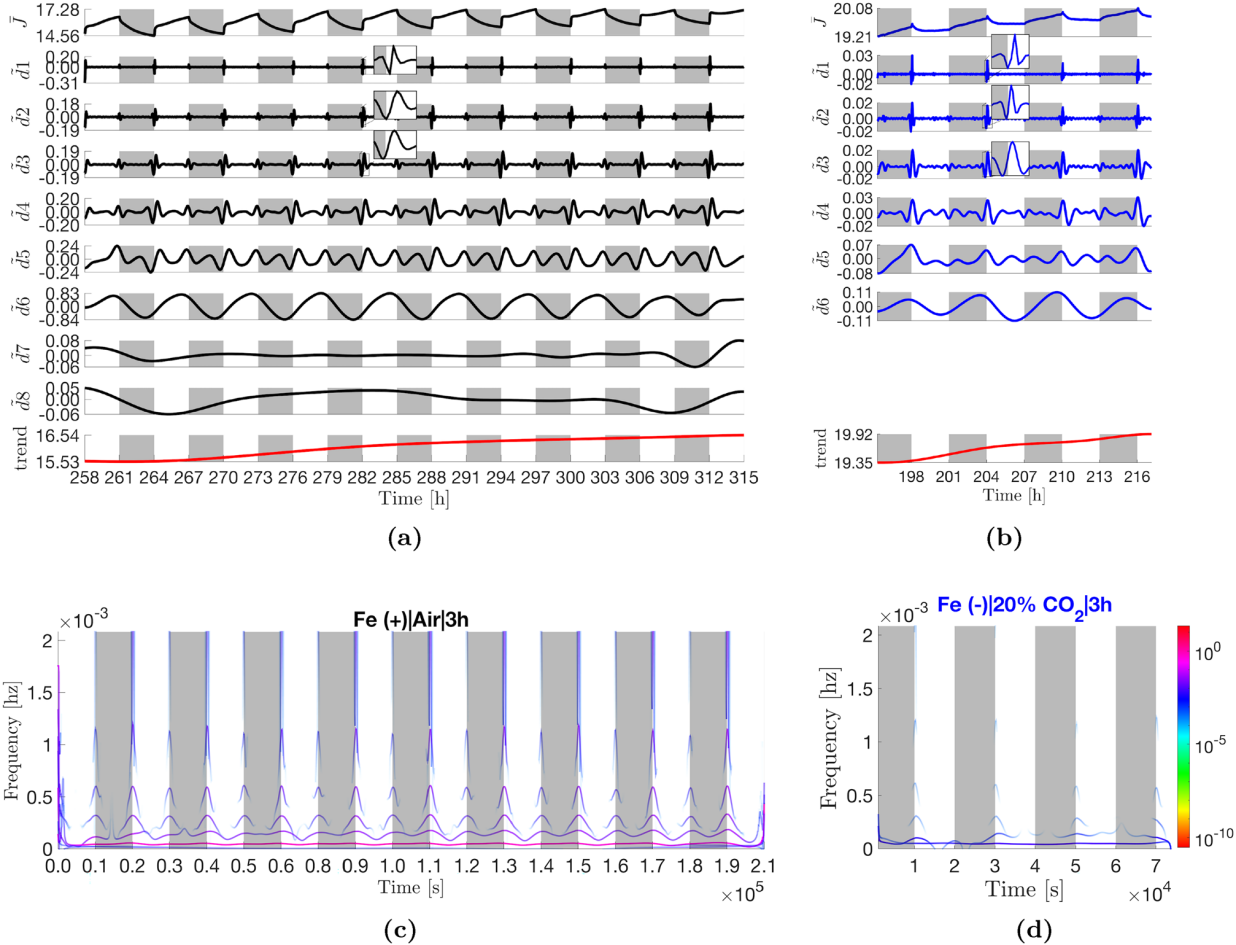
IMFs and Hilbert spectra of Fe(+)|Air|3 h and Fe(−)|20% CO${_2}$|3 h after replenishment of respective media.

Each of the profiles in Fig. 2, were decomposed using the ICEEMDAN algorithm (see Algorithm 2 under “Methods”). In addition, because (i) the current profiles are entrained to a square wave (the on/off light pattern) and (ii) EMD-based algorithms are known to be sensitive to the delta or step functions, ICEEMDAN was carried out on synthetic square waves (abbreviated as SW and shown in purple in graphs) of the same sampling frequency and similar magnitude as the empirical data to get a better understanding of how the algorithm performs at the discontinuous points. The resulting intrinsic mode functions are displayed in Figs. 3 and 6a,b. The Hilbert transform was then performed on all the extracted IMFs to compute the instantaneous frequencies and energies. The data from the computed analytic signals can be displayed as frequency profiles contoured with instantaneous energies such as displayed in Fig. 4 for the Fe(−)|Air|3h condition. When plotted on a single axis, the contoured instantaneous frequency profiles form the Hilbert spectrum. The Hilbert spectra for the different operating conditions are shown in Figs. 5 and 6c,d. In addition, the IMFs and Hilbert spectra of the corresponding abiotic, media-only devices are shown in Supplementary Figs. S12–13 to identify the background. For some modes, both the peak-to-peak duration and median periodicity (computed from the inverse of the median instantaneous frequency shown in Fig. 5) are quoted. Reason for discrepancies in the two numbers is addressed in the Discussion section of the paper. Decomposition of the synthetic square waves is reported first, with the rest of the decompositions reported in relation this.

In the 3 h:3 h light regime, the synthetic square wave decomposes into 7 IMFs (Fig. 3a). IMFs $\tilde{d}1-4_{|3h}$ contain a series of waveforms at discontinuous points (where there is a light change) that are reminiscent of cubic spline wavelets, and zero otherwise. The waveforms decrease in frequency as IMF number increases (4.16, 3.12, 2.50, and 0.52 mHz for $\tilde{d}1-4_{|3\;h}$ in that order) (Fig. 5a). IMF $\tilde{d}5_{|3\;h}$ is a frequency modulated (FM) mode with a repeating pattern of three peaks during a period, the first approximately 0.4 h after dawn, the second 2.2 h later (or 0.4 h before dusk) and the last occurring in the night 1.9 h after the second. IMF $\tilde{d}6_{|3\;h}$ oscillates in a sinusoidal manner with the same period as the light-dark pattern (6.0 h or 0.046 mHz). The mode peaks 6.0 h after dawn, and is minimum 6 h after dusk. Lastly, IMF $\tilde{d}7_{|3h}$ is a low-energy mode with with a period of 12.0 h (or 0.023 mHz).

In the 12 h:12 h regime, the synthetic wave decomposes into 9 IMFs (Fig. 3e). Modes $\tilde{d}1-6_{|12\;h}$ contain waveforms at discontinuous points with frequencies of 4.15, 3.36, 2.57, 2.30, 2.15, and 0.42 mHz for $\tilde{d}1-6_{|12\;h}$, in that order. The introduction of the two new modes $\tilde{d}5-6_{|12\;h}$ and the change in frequency of $\tilde{d}4{}_{|12\;h}$ relative to the 3 h:3 h regime may be due to the much longer periods (4 $\times$ longer). IMF $\tilde{d}7_{|12\;h}$ is an FM mode with three peaks during a period, analogous to $\tilde{d}5_{|3\;h}$. The first peak is 1.5 h after dawn, the second 9.1 h later (1.4 h before dusk), and the third 7.5 h later in the night. Mode $\tilde{d}8_{|12\;h}$ is analogous to mode $\tilde{d}6_{|3\;h}$ in that it oscillates in a sinusoidal manner with the same frequency as the light-dark pattern (0.012 mHz or 24 h period) with a peak and trough in the middle of the day and night respectively. Lastly, $\tilde{d}9_{|12\;h}$ is a low-energy, low-frequency mode analogous to $\tilde{d}7_{|3\;h}$ that oscillates with a period of 36.5 h (or 0.008 mHz).

#### Eight modes are extracted from the control current density profile

The Fe(+)|Air|3 h current density profile deviates the most from a pure square wave (i.e., the least steep transitions during light changes) and also has the lowest amplitude. Eight IMFs were extracted from the profile (Fig. 3b). Equivalent waveforms at the discontinuous points of $\tilde{d}1-4_{|3\;h}$ as seen in the synthetic decomposition are present but masked by noise due to their low amplitudes. It will be shown later that the amplitude of the waveforms increase as the profile becomes more ’square’ and increases in amplitude with time. IMF $\tilde{d}5_{|3\;h}$ is analogous to the equivalent mode in the synthetic square wave decomposition. Mode mixing is visible at the beginning of the time series between $\tilde{d}5_{|3\;h}$ and $\tilde{d}6_{|3\;h}$ (0–3 h); additional sifting operations and increased number of ensembles failed to eliminate the phenomenon. Different from the synthetic wave decomposition, the mode is both frequency and amplitude modulated (FM-AM) and has altered peak timings. Amplitude modulation is exhibited by a repeating pattern of peak heights: the first peak (here 1.0 h instead of 0.4 h after dawn) is the highest, the second peak (here 1.51 ± 0.04 h after the first vs. 2.2 h) is slightly lower, and the third peak (here 2.17 ± 0.14 h after the second vs. 1.9 h) is the lowest. IMF $\tilde{d}6_{|3\;h}$ oscillates with a peak-to-peak time span of 5.9 ± 0.04 h and a median period of 6.0 h. The mode peaks around 0.6 h (35 *min*) before dusk (in comparison, the equivalent mode in the synthetic decomposition peaks exactly in the middle of the day). Lastly, IMF $\tilde{d}7_{|3\;h}$ oscillates with a period of approximately 16.1 h (vs. 12 h in the synthetic decomposition) and a new mode, $\tilde{d}8_{|3\;h}$, oscillates with a period of 22.2 h.

#### Modes extracted from the more square Fe(−) profile closer resemble modes extracted from the synthetic decomposition

The Fe(−)|Air|3 h current density profile is visibly more ‘square’ than the Fe(+)|Air|3 h profile (steeper transitions, flatter profile in the day). The profile expanded to eight IMFs (Fig. 3c). Waveforms are visible at the discontinuous points in IMFs $\tilde{d}1-4_{|3\;h}$ (Fig. 4). These waveforms have substantially lower frequencies compared to the square wave decomposition (2.4, 1.2, 0.6, and 0.3 mHz for $\tilde{d}1-4_{|3\;h}$, in that order) (Fig. 5c). This is expected given the transitions during the light changes are not as steep as the delta function. Like the control, mode $\tilde{d}5_{|3\;h}$ also exhibits both frequency and amplitude modulation. However, the timings of the three peaks are less altered relative to the equivalent mode in the synthetic decomposition. Under iron starvation, the early day peak occurs 0.6–0.7 h after dawn (*vs*. 0.4 h) but the peak-to-peak timespans are unchanged: 2.19 ± 0.02 h (1st to 2nd peak) and 1.89 ± 0.02 h (2nd to 3rd peak). Mode $\tilde{d}6_{|3\;h}$ had a 6.0 ± 0.1 h peak-to-peak timespan and 6.0 h median period with peaks occurring in the middle of the day as in the equivalent mode of the synthetic square wave (although appear phase shifted by approximately $\pi /4$ rads towards dawn relative to the control). Finally, $\tilde{d}7-8_{|3\;h}$ have periods of 16.1 h and 33.2 h, respectively, the latter significantly longer relative to the control (+11 h).

#### All modes exhibit an apparent $\pi$ rad phase shift and reduced amplitudes in the presence of 20% CO${_2}$

The Fe(−)|20% CO${_2}$|3 h current density profile expanded to eight IMFs (Fig. 3d). The waveforms of $\tilde{d}1-4_{|3\;h}$ have approximately the same frequencies as their respective Fe(−)|Air|3 h analogs, albeit generally lower energies than the latter (Fig. 5d). As expected from the current density profile (Fig. 2c), all IMFs have a $\pi$ rad phase shift relative to the control, most clearly exemplified in $\tilde{d}6_{|3\;h}$ The FM-AM mode $\tilde{d}5_{|3\;h}$ has similar peak-to-peak timespans as its Fe(−)|Air|3 h analog, 2.14 ± 0.03 h (1st to 2nd peak) and 1.93 ± 0.13 h (2nd to 3rd peak). Mode $\tilde{d}6_{|3\;h}$ has a peak-to-peak timespan of 6.0 ± 0.4 h and a median period of 6.0 h. Modes $\tilde{d}7-8|_{3\;h}$ oscillate with longer periods than their control analogs at 25 h and 27 h, respectively. However, there is uncertainty due to an abnormally prolonged decline in current density between 48–51 h of operation that caused mode mixing (see “Methods” for explanation) across $\tilde{d}5-8_{|3\;h}$ (visible from 48–54 h).

#### Waveforms appear over time in the Fe(+) IMFs as the profiles become more square

There are some notable differences in the control (Fe(+)|Air) IMFs extracted from the 12 h:12 h current density profiles compared to the 3 h:3 h regime as the underlying current density profile became more square, with steeper gradients at the light changes. First, there is a large increase in the amplitudes of all oscillations in the Fe(+)|Air|12 h IMF set, Figs. 3f and 5f. Second, the waveforms characteristic in modes $\tilde{d}1-4_{|12\;h}$ of a square wave decomposition become visible. The waveforms have the same frequencies as those in Fe(−)|Air|3 h, but with significantly larger amplitudes particularly at dawn (Fig. 5c,f), which coincides with the greater photoresponse of the Fe(+)|Air|12 h profiles. In addition, modes, $\tilde{d}5-6_{|12\;h}$ (absent in 3 h:3 h regime) have waveforms of lower frequency than their synthetic equivalents ($\approx$ 0.16 and 0.09 mHz vs. 2.15 and 0.42 mHz). Of note, the amplitudes of the $\tilde{d}1-4_{|12\;h}$ (and $\tilde{d}5-6_{|12\;h}$) waveforms diminish with time which was not observed in the Fe(−)|Air|3 h condition. Wavelets in IMF $\tilde{d}1_{|12\;h}$ oscillations peak at dawn, while in $\tilde{d2}-6_{|12\;h}$, the peaks are increasingly shifted to the left of dawn, with $\tilde{d}4-6_{|12\;h}$ having minima coincident with the light transition or to the left of dawn, insets Fig. 3f. The superposition of $\tilde{d}1-3_{|12h\;}$ is thus revealed as responsible for the dip in current density observed immediately after illumination, inset Fig. 2d. The decreasing amplitudes of the oscillations with each successive dawn is accountable for both the shallower dip and the diminishing gradient of the initial photoresponse from period-to-period.

#### The sinusoidal mode $\tilde{d}8_{|12\;h}$ peaks near dusk rather than the middle of the day in the control

IMFs $\tilde{d}7_{|12\;h}$ and $\tilde{d}8_{|12\;h}$ are analogs of the FM-AM $\tilde{d}5_{|3\;h}$ and the constant frequency $\tilde{d}6_{|3\;h}$ in the 3 h:3 h regime, respectively. There is however significant mode mixing between the two modes, particularly at the beginning of the time series where it is exacerbated by edge effects (*i.e.* upper and lower envelopes are fit using splines which may have poorer fit at the beginning and end of the time series). The mixing was mitigated to some extent by increasing the signal-to-noise ratio and number of ensembles during the decomposition (see “Methods”) but could not be fully eliminated. Peak-to-peak timespans for mode $\tilde{d}7_{|12\;h}$ were $\approx$ 6.04 ± 0.16 h (1st to 2nd peak) and $\approx$ 8.68 ± 0.56 h (2nd to 3rd peak) compared to 9.1 h and 7.5 h for the equivalent modes in the square wave decomposition, respectively. Mode $\tilde{d}8_{|12\;h}$ oscillates with peak-to-peak duration of 24.0 ± 1.1 h and a median periodicity of 25.8 h (vs. 24.0 h for the equivalent synthetic square wave mode). The mode peaks about 2.0–2.5 h before dusk (vs. in the middle of the day in its synthetic analog). IMF $\tilde{d}8_{|12\;h}$ appears to be the main contributor to the second gradient of the two-sloped photoresponse seen in the current density profile. Over time (162–234 h), the amplitude of the oscillation decreases accounting for the dwindling photocurrent observed in Fig. 2d. Interestingly, modes $\tilde{d}7-8_{|3\;h}$ from the Fe(+)|Air|3h decomposition, seemingly disappear when BPVs are operated under a 12 h:12 h light-dark pattern. It is plausible that energy of mode $\tilde{d}8_{|3\;h}$ (0.013 mHz) is embedded into mode $\tilde{d}8_{|12\;h}$ (0.012 mHz) since they are close in frequency.

#### Iron stress shortens the periods of modes $\tilde{d}7-8_{|12\;h}$ by 1.2 h under 12 h:12 h light-dark periodicity

The Fe(−)|Air|12 h profile is visually more square than the Fe(+)|Air|12 h due to a flatter profile in the day and a steeper decay in current in the night (Fig. 3g). Thus the IMFs extracted from the Fe(−)|Air|12 h profile were more in line with those of the square wave decomposition. The peak-to-peak timespans of the FM-AM $\tilde{d}7_{|12\;h}$, 8.61 ± 0.36 h (1st to 2nd peak) and 7.43 ± 0.15 (2nd to 3rd peak), are roughly equal to the square wave modes within uncertainty. In describing the Fe (−)|Air|12 h IMFs relative to the control, iron stress caused a phase shift such that the peaks advance towards dawn which is most visible in $\tilde{d8_{|12\;h}}$. Notably, the duration between $\tilde{d}7_{|12\;h}$ dusk-timed and nighttime peaks, as well as the peak-to-peak duration and median periodicity of $\tilde{d}8_{|12\;h}$ (23.1 ± 1.1 h and 24.6 h, respectively) are shortened by 1–1.2 h relative to the control. The duration between $\tilde{d}7_{|12\;h}$ early day and dusk-timed peaks increases by $\approx 2.5$ h due to an advance of the early day peak towards dawn under iron stress. In $\tilde{d}1_{|12\;h}$, the phase shift results in the minima, rather than the maxima, of oscillations occurring at dawn, inset Fig. 3g. The absence of a current dip after illumination in the Fe(−)|Air|12 h BPVs is attributable to this change.

#### Amplitude changes in the modes are correlated with ferric ion concentration in the media

In comparing Fig. 3f,g, it can be seen that, the amplitudes of $\tilde{d1}-8_{|12\;h}$ oscillations in the control diminish across successive periods until, at 234 h, they reach similar magnitudes as their Fe(−)|Air|12 h analogs. Given that the only difference in the two conditions was the initial ferric ion concentration in the respective growth media, the results hint that the decaying amplitudes are a function of gradual ferric ion depletion in the Fe(+)|Air medium. To corroborate this conclusion, amplitudes of $\tilde{d}1-4_{|12\;h}$ analogs, $\tilde{d}1-4_{|3\;h}$, were observed to be ferric-sensitive. That is, when the depleted media in the Fe(+)|Air BPVs were replenished with fresh BG11 at $\approx$ 246 h and the BPVs operated under a 3 h:3 h light-dark period (Fig. 6a), the modes exhibited period-to-period recoveries in amplitude until the 294 h dawn, before decaying again, Fig. 6a. In addition, the $\tilde{d}5_{|3\;h}$ peak-to-peak timespans also evolve to match those of the Fe(−)|Air|3 h condition, 2.15 ± 0.07 h (1st to 2nd peak) and 1.78 ± 0.09 h (2nd to 3rd peak). Mode $\tilde{d}8_{|12\;h}$ is the exception to this. The amplitude of $\tilde{d}8_{|12\;h}$ decreased to an apparent minimum and remained relatively constant in amplitude from 258 h onwards, Fig. 3f and $\tilde{d}6_{|3\;h}$ in Fig. 6a.

#### Positive light response in the presence of CO${_2}$ is due the four highest frequency modes

As in the 3 h:3 h regimes, the presence of 20% CO${_2}$ caused a $\pi$ rad phase shift in the Fe(−)|20% CO${_2}$|12 h IMFs relative to the 12 h:12 h control (Fig. 3h). The oscillations also exhibited the diminished $\tilde{d1}-7_{|12\;h}$ amplitudes observed under iron depletion. Mode $\tilde{d}8$ had a peak-to-peak timespan of 24.3 ± 0.7 h and a median period of 24.4 h. With the exception of $\tilde{d}8_{|12\;h}$, amplitudes were even lower than their Fe(−)|Air|12 h analogs. Relative to $\tilde{d}1-4_{|3\;h}$ (Fe(−)|20% CO${_2}$|3 h (Fig. 3d), small phase differences are visible in $\tilde{d}1-4_{|12\;h}$ that push the peaks of the waveforms just to the right of dawn, insets Fig. 3f. A period-to-period increases in amplitudes of the modes is also visible. With these changes, it becomes evident that the superposition of $\tilde{d}1-4_{|12\;h}$ form the short-lived positive light response observed beginning from the 114 h dawn, inset Fig. 2f. Under a 12 h:12 h light-dark period, the peak-to-peak timespans of the FM-AM $\tilde{d}7_{|12\;h}$ are 8.17 ± 0.14 h (1st and 2nd peaks) and 8.02 ± 0.18 (2nd and 3rd peaks).

#### Iron stress increases the basal exoelectrogenic activity

The effect of iron starvation is most pronounced in the residuals, i.e. the trends, Fig. 3b–d,f–h. In the early stages of operation ($<80$ h), there was an up to 5.7-fold increase in the magnitude of the residual under iron depleted conditions, which declines to approximately 1.8-fold in the later stages (> 162 h) of operation as ferric ions are also depleted in the control BPVs. The result that iron starvation increases the basal exoelectrogenic activity in *S. elongatus* is consistent with previous results reported by the research group^7^.

#### Spread of energy is concentrated in the frequency of the light pattern

**Figure 7:**
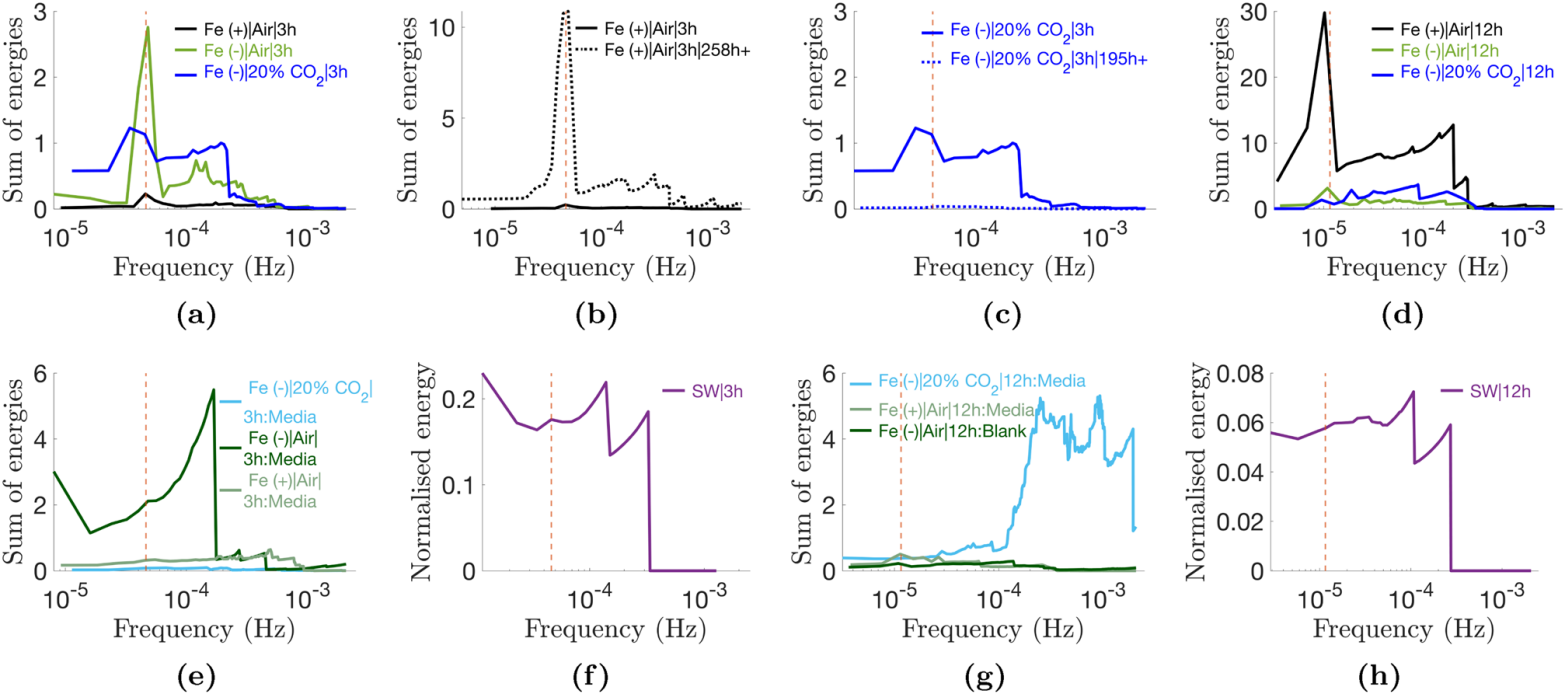
Marginal spectra. The marginal spectra show the spread of variable energies (i.e, excluding the trend) across the frequency range as calculated by Eq. (10). The energy of the trend overpowers the variable energies and is therefore excluded. The dotted lines shows the frequency of the light-dark pattern: 0.046 mHz and 0.012 mHz for the 3 h:3 h and 12 h:12 h light-dark periodicities, respectively.

The biggest difference between the decomposition of the synthetic square waves and the experimental data is the spread of variable energy across the different modes. It is seen that in the experimental data, the energy is concentrated at the frequency of the light-dark pattern to which the current density profile is entrained (Fig. 7a–d). Differently, in the synthetic square wave, the variable energy is concentrated at higher frequencies of the waveforms that occur at discontinuous points (Fig. 7f,h). Notably, the blank abiotic devices have marginal spectra more akin to that of the synthetic square waves (Fig. 7e,f).

## Discussion

In the following discussion, initial hypotheses on the physical phenomena that lead to deviations of the modes from those obtained from the decomposition of synthetic square waves are developed for future investigations. The first clue available in the results in the 25.8 h median period length computed for $\tilde{d}8_{|12\;h}$ (control) which is identical to the 25.6 ± 0.06 h circadian free running period (FRP) period of *S. elongatus* measured at a light intensity of 25 $\upmu$mol $m^{-2}$ $s^{-1}$^27^. In *S. elongatus*, nearly all genes have been found to be expressed rhythmically under the control of a circadian (daily) clock^28^. Thus, most biochemical processes in cyanobacteria have a natural diurnal periodicity^28,29^. This alludes to a link between exoelectrogenesis and the circadian clock in cyanobacteria. Hypotheses are developed that are consistent with the state of the art model of the cyanobacteria circadian system, putative exoelectrogenic pathways, and the empirical data collected in this work. To give background to the discussion, the circadian system in *S. elongatus* is briefly presented first. In this section, the subscripts $|3\;h$ and $|12\;h$ are dropped for $\tilde{d}1-4$ since these modes have the same frequencies in both the 3 h:3 h and 12 h:12 h light-dark regimes.

### Overview of the cyanobacteria circadian clock

The circadian system is composed of input pathways, a central biochemical clock and output pathways^28,29^. In cyanobacteria, the key input pathways are CikA (circadian input kinase A) and the redox-active protein LdpA (light-dependent period A). The central biochemical clock is the KaiABC protein complex. The output pathway that transmits temporal information from the biochemical clock includes the kinase *Synechococcus *adaptive sensor A (SasA) and the transcription factor regulator of phycobilisome association A (RpaA), which together control gene expression, timing of cell division and other rhythmic biological activity. The KaiABC protein complex keeps time through a highly ordered, sequential cycle of phosphorylation by ATP (day) and dephosphorylation to ADP (night) over a diurnal period^28^. The cycle is independent of the cell division rate. The biochemical clock identifies day and night via the redox state of the plastoquinone pool in the photosynthetic electron transport chain. During the day, the plastoquinone pool is reduced by electrons from upstream photosystem II (PSII), but is rapidly oxidised when light is switched off. CikA and KaiA can only bind to oxidised quinones, and in turn, CikA interacts to KaiC only when bound to an oxidised quinone, signalling the onset of night. Secondly, KaiC senses changes in the ATP/(ATP + ADP) ratio which decreases gradually during the dark interval (irrespective of when the light goes off), signalling the length of night in a light-dark period. The second input component, LdpA, is an iron-sulfur centre redox-active protein that senses changes to the electron transport chain that are dependent on light intensity. Temporal information from the biochemical clock is relayed to gene expression via SasA/RdpA^28,30^. During the day, SasA binds to KaiC promoting SasA autophosphorylation followed by phosphotransfer to RdpA to form RdpA-P. RdpA-P is the active form of RdpA and is accumulated over the course of the day, peaking at the day-night transition (dusk). RdpA-P binds directly to about 100 targets in the *S. elongatus* genome, activating dusk-peaking class 1 genes, while repressing the formation of dawn-peaking class 2 genes. Over the course of the night, CikA removes the phosphoryl groups from RdpA-P. In addition to RpaA, there is evidence that the transcription regulator RpaB, which regulates gene expression in response to environmental stress conditions such as photo, thermal or oxidative stress, also plays a part in the output pathway by working cooperatively with RpaA. RpaB can inhibit the phosphorylation of RpaA^31^. Oscillations in the phosphorylation of RpaB are however only present when cells are grown under a 12 h:12 h light-dark period^28^.

### Inverse of the instantaneous frequency is free running period, while peak-to-peak duration is entrained to light-dark period

It is suggested that the 25.8 h median period computed from the inverse of the median instantaneous frequency for mode $\tilde{d}8_{|12\;h}$ reflects the free running period of *S. elongatus* circadian rhythms. The shorter 24 h peak-to-peak duration results from a reset of the oscillation initiated by the dark pulse after 12 h of light. The dark pulse interrupts the natural trajectory of the oscillation and entrains the peak-to-peak duration to the light-dark period.

### The relative frequencies of waveforms in modes $\tilde{d}1-4$ and $\tilde{d}5-6_{|12\;h}$ may hold implicit information on the relative diversion of PETC electrons across the different conditions

The lower frequency of the cubic spline waveforms in $\tilde{d}1-4$ and $\tilde{d}5-6_{|12\;h}$ compared to their equivalents in the square wave decomposition reflects the delay between light switching on/off and the biochemical activation/deactivation of photo-driven exoelectrogenesis (i.e. electron generation in the PETC, intracellular diffusion of redox species and extracellular electron export). Further, it was demonstrated in the results that impersistent modes $\tilde{d}1-4$ are ferric sensitive, i.e., their amplitudes decay and recover in response to ferric ion concentration. The behaviour correlates to pigment changes under iron starvation (Supplementary Fig. S3) aimed at retrenching the PETC that are known to be reversible upon iron replenishment^32,33^. Therefore, it is is speculated that the relative amplitudes of the waveforms across the different conditions may be used to quantify the relative number of electrons diverted from the PETC to exoelectrogenesis.

### Shorter peak-to-peak durations and median periods under iron stress are thought to be due to retrenchment of LdpA

Mode $\tilde{d}8_{|12\;h}$ oscillated with median periods of 25.8 h, 24.6 h and 24.3 h in the Fe(+)|Air|12 h, Fe(−)|Air|12 h and Fe(−)|20% CO${_2}$|12 h conditions respectively, compared to a 24 h period of the equivalent mode in the synthetic square wave decomposition. This is equivalent to stating that the mode had periods that were 1.2–1.4 h shorter than the control in the Fe(−)|Air|12 h and Fe(−)|20% CO${_2}$|12 h conditions. Further the mean peak-to-peak duration was 1 h shorter than the control in the Fe(−)|Air|12 h condition. Similarly, the duration between the dusk-timed peak and lower nighttime peak in $\tilde{d}7_{|12\;h}$ was found to be $\approx$ 1.2h shorter under iron limitation. During culture growth, Fe(−)|Air and Fe(−)|20% CO${_2}$ cells exhibited a significant drop in chlorophyll *a* content, as well as substantial blue shifts in absorption spectra over time, particularly the 633 nm (phycocyanin) and 683 nm (chlorophyll *a*) absorption peaks (Supplementary Fig. S3). The pigment changes were more severe in the presence of 20% CO${_2}$. These are well-documented characteristic responses to iron starvation in *S. elongatus* that are part of a broader rearrangement of the photosynthetic system, including reducing the amount of iron/iron-sulfur rich photosynthetic proteins^27,32,34,35^. Interestingly, mutant cells lacking the gene encoding the LdpA protein exhibit these same phenotypic changes, namely: oscillatory periods that are one hour shorter due to insensitivity to light gradients (i.e., the circadian period length does not increase with reducing light intensity as per Ashcoff’s rule); and reduced phycocyanin content^27,28^. Since LdpA also contains an iron-sulfur core, it is suggested that the shorter $\tilde{d}8_{|12\;h}$ and $\tilde{d}5_{|3\;h}$/$\tilde{d}7_{|12\;h}$ periods observed in the iron depleted conditions are the result of retrenchment of the protein as part of the ensemble of adaptations to reduce iron demand.

### Introduction of amplitude modulation in $\tilde{d}5_{|3\;h}$/$\tilde{d}7_{|12\;h}$ thought to be linked to *psbAI* circadian expression

Two key differences are observed in the in modes $\tilde{d}5_{|3\;h}$/$\tilde{d}7_{|12\;h}$ compared to the equivalent modes extracted from the synthetic square wave: (i) amplitude modulation is introduced, in particular, the second (dusk) and third (nighttime) peaks were lower than the first (early day), with the third lower than the second; and (ii) in the control, the peak-to-peak duration from the 2nd to 3rd peaks was altered to 8.68 ± 0.56 h (vs. 7.5 h). Strikingly, the altered timings and amplitude ratios of the second and third peaks are reminiscent of the expression rhythm of the photosynthesis gene *psbAI.* The gene encodes for the D1 reaction centre protein of PSII, in particular the D1:1 form, that plays a key role in the initiation of photosynthesis. At a light intensity of 15 $\upmu$mol m$^{-2}$ s$^{-1}$, the gene is expressed with two peaks within a diel period: a higher dusk peak and a lower nighttime peak ca. nine hours later^36^. It is therefore hypothesised that amplitude modulation in $\tilde{d}5_{|3\;h}$/$\tilde{d}7_{|12\;h}$ oscillations is introduced from changes in electron flow linked to the circadian expression of *psbAI.*

### Diurnal intracellular glycogen fluctuations speculated to push day peak in mode $\tilde{d}6_{|3h}$/$\tilde{d}8_{|12h}$ off-centre

In the control, modes $\tilde{d}6_{|3\;h}$/$\tilde{d}8_{|12\;h}$ peaked approximately 80% through the day (0.6 h and 2–2.5 h before dusk, respectively) compared to the middle of the day in the equivalent modes of the synthetic square wave decompositions. *S. elongatus* intracellular glycogen levels oscillate under circadian control, increasing over the course of the day and peaking near dusk (in standard laboratory conditions)^37,38^.Additionally, it is known that *S. elongatus* increase glycogen stores under iron limited conditions^33^. The need to maintain glycogen stores may limit the amount that is depleted in the night and thus accumulated during the day, which would explain the lower but constant amplitude of $\tilde{d}6_{|3h}$/$\tilde{d}8_{|12h}$ in the Fe(−) and older Fe(+) BPVs. Combined, the studies offer a coherent conceptual model for a glycogen-coupled electron flux dragging the peaks off-centre, with a circadian rhythm that peaks near dusk and has a lower *variation*in iron depleted conditions. Variation is emphasised here since increase in intracellular glycogen content is known to increase exoelectrogenic activity as noted in the discussion of the trend below.

### Phase shifts under iron stress and in the presence of CO${_2}$ hypothesised to be due to changes to intracellular ATP/(ATP + ADP) ratio

In this paragraph, changes in modes $\tilde{d}6_{|3\;h}$/$\tilde{d}8_{|12\;h}$ and $\tilde{d}5_{|3\;h}$/$\tilde{d}7_{|12\;h}$ are discussed relative to the control. In vitro and in vivo experiments have shown that when the intracellular ATP/(ATP + ADP) ratio (%ATP henceforth) is reduced below normal (typically $\approx$ 80% in the light, falling to $\approx$ 50% within 2–3 h of darkness), circadian rhythms advance towards dawn^37,39^. The more extreme the reduction in %ATP during night, the greater the phase advance after a dark interval. Thus, the advancement of modes $\tilde{d}6_{|3\;h}$/$\tilde{d}8_{|12\;h}$ and $\tilde{d}5_{|3\;h}$/$\tilde{d}7_{|12\;h}$ (early day) peaks towards dawn in the Fe(−)|Air condition is hypothesised to be caused by lower intracellular %ATP in the cells at night. This is consistent with lower photosynthetic activity, ergo reduced accumulation of ATP, during the day under iron limitation.

The phase shifts observed in the Fe(−)|20% CO${_2}$ modes were more extreme. There was evidence that the presence of CO${_2}$ adversely affected the functioning of the CikA protein that is responsible for cleaving the phosphorous group from Rpa-P during the night. Fe(−)|20% CO${_2}$ cells, were found to be elongated by up to 1.6 $\upmu$m relative to Fe(−)|Air cells (Supplementary Fig. S5) and by $\approx$ 0.5–0.8 $\upmu$m relative to cells grown in standard conditions (reported in previous work)^25^. In *S. elongatus,* this phenotype indicates elevated levels of RpaA-P, which implies a reduction/interruption in normal CikA activity^28,31^. Fe(−)|Air cells exhibited the opposite phenotype, with shortened cells that were only 62–80$\%$ the length of iron replete cells (Supplementary Fig. S5). Cells lacking CikA overaccumulate glycogen during the day, and exhibit higher %ATP than wild-type cells over the course of night^37^. Consequently, the mutant cells exhibit reduced ability to reset phase following a dark pulse. It is therefore speculated that the elevated levels of CO${_2}$ increase nighttime intracellular %ATP thus causing the peculiar phasing of oscillations observed in the Fe(−)|20% CO${_2}$ modes.

### Magnitude the trend is linked to bioavailability of iron

The trend was the only component to exhibit a larger magnitude in iron-depleted conditions. The implication then, is that the biochemical process responsible for the trend is linked to an iron induced accumulation of a metabolite or the over-expression of a protein that participates in the exoelectrogenic pathway. Of the suite of iron starvation induced adaptations in *S. elongatus*, increase in flavodoxin, expression of iron starvation induced outer membrane proteins, and increase in glycogen content are the most relevant suspects consistent with putative exoelectrogenic pathways.

To reduce iron demand in iron depleted conditions, levels of the redox protein flavodoxin increase in replacement of the retrenched electron mediator ferrodoxin^34^. However, since flavodoxin, like ferrodoxin, is an intracellular electron carrier yet to be implicated in electron export, there no evidence to support that replacement of one with the other alters exoelectrogenic rates.

Three outer-membrane proteins of 92, 35 and 48–50 kDa mass are induced in iron starved *S. elongatus* that are otherwise unexpressed in cells grown in iron replete media^40^. Further, as highlighted in the introduction, cyclic voltammetry experiments in the research group provided evidence of distinct outer membrane redox activity in iron starved cells which correlated with the up-regulation of IrpA (iron-regulated protein A, 38.6 kDa) in the cytoplasmic membrane, SomB1 (outer membrane porin) in the outer membrane, and a proposed c-type cytochrome IrpB (49.3 kDa)^7^. Taken together, the evidence could support a hypothesis that the magnitude of the trend is linked to one or more of the iron starvation induced proteins.

Lastly, current output has been reported to be proportional to intracellular glycogen content in *Synechococcus *sp. (strain not declared) BPVs operated in the presence on 3% v/v CO${_2}$^41^. As noted earlier, intracellular glycogen content increases under iron starvation. Thus, the larger magnitude of the trend under iron stress is consistent with known changes in intracellular glycogen content, as well as the known effect of intracellular glycogen levels on current generation by cyanobacteria.

### Combining ICEEMDAN with machine learning is a promising approach to modelling photobioelectrochemical systems

One of the key motivations for this work was to decompose BPV current density into more regular, physically meaningful subcomponents that can be modelled with machine learning or other modelling approaches. It is evident that ICEEMDAN achieves this objective. Each current density profile is decomposed into a maximum of eight modes that are predictable and, individually, substantially easier to model than the parent observed current density. Mode $\tilde{d}6_{|3\;h}$/$\tilde{d}8_{|12\;h}$ for example, is easily modelled with a simple sin/cosine function. As the physical meanings of the modes are unravelled, for example if the proposed link between ferric ion concentration and the attenuation of amplitude or that between intracellular %ATP and phase shifts are confirmed, simple but robust models can be developed that take as input forecasts of the environmental nutrient and intracellular metabolite concentrations to model individual IMFs, which can then be summed to provide a prediction of BPV performance over time. A combination of empirical mode decomposition and LSTM networks has been recently used to successfully model complex signals in nuclear power plants^42^.

Table 1 summarises the results and hypothesis presented and discussed.

**Table 1:** Summary of frequency bands obtained via the Hilbert–Huang transform and hypotheses for their physical meaning. The Fe(−) effect column shows the response to iron-depleted conditions. In addition, all IMFs exhibited some form of a phase shift relative to the control under iron-depleted conditions, which was made more severed in a 20% CO${_2}$ atmosphere.

IMF	SW (mHz)	$f_{min}$ (mHz)	$f_{max}$ (mHz)	$f_{mid}$ (mHz)	$f_{mid}/f_{I_{v}}$ (–)	Fe(−) effect	Hypothesis
**12 h:12 h light-dark period (**$f_{I_{v}}=0.012$** mHz)**							
$\tilde{d}1_{|12\;h}$	4.150^a^	2.100	2.400	2.250	194	Lower amplitude	Tied to activation of PETC
$\tilde{d}2_{|12\;h}$	3.355^a^	1.140	1.180	1.160	100	Lower amplitude	Tied to activation of PETC
$\tilde{d}3_{|12\;h}$	2.573^a^	0.550	0.600	0.575	49.7	Lower amplitude	Tied to activation of PETC
$\tilde{d}4_{|12\;h}$	2.299^a^	0.280	0.320	0.300	25.9	Lower amplitude	Tied to activation of PETC
$\tilde{d}5_{|12\;h}$	2.146^a^	0.155	0.158	0.157	13.5	Lower amplitude	Tied to activation of PETC
$\tilde{d}6_{|12\;h}$	0.424^a^	0.076	0.086	0.081	7.00	Lower amplitude	Tied to activation of PETC
$\tilde{d}7_{|12\;h}$	0.031^b^	0.046	0.031	0.039	3.33	− 1 *h* period	Tied to *psbAI* expression
$\tilde{d}8_{|12h}$	0.012^b^	0.012	0.012	0.012	1.00	− 1 *h* period	Tied to glycogen levels
						Higher amplitude	
$\tilde{d}9_{|12\;h}$	0.008^b^	NM	NM	NM	NM	NM	NM
**3 h:3 h light-dark period (**$f_{I_{v}}=0.046$** mHz)**							
$\tilde{d}1_{|3\;h}$	4.158^a^	2.100	2.400	2.250	48.6	Lower amplitude	Tied to activation of PETC
$\tilde{d}2_{|3\;h}$	3.117^a^	1.140	1.180	1.160	25.1	Lower amplitude	Tied to activation of PETC
$\tilde{d}3_{|3\;h}$	2.501^a^	0.550	0.600	0.575	12.4	Lower amplitude	Tied to activation of PETC
$\tilde{d}4_{|3\;h}$	0.524^a^	0.280	0.320	0.300	6.48	Lower amplitude	Tied to activation of PETC
$\tilde{d}5_{|3\;h}$	0.182^b^	0.185	0.123	0.154	3.33	Lower amplitude	Tied to *psbAI* expression
$\tilde{d}6_{|3\;h}$	0.046^b^	0.046	0.046	0.046	1.00	Higher amplitude	Tied to glycogen levels
$\tilde{d}7_{|3\;h}$	0.023^b^	0.017	0.017	0.017	0.37	No change	Inconclusive
$\tilde{d}8_{|3\;h}$	NM	0.013	0.013	0.013	0.27	+ 11 *h* period	Inconclusive
**Universal**							
Trend	NM	NM	NM	NM	NM	1.8–5.7 $\times$ larger	Magnitude tied to Fe levels

*SW* square wave,

$f_{min}$ minimum of IMF frequency band, $f_{max}$ maximum of IMF frequency band, $f_{mid}$ middle frequency of band, $f_{I_{v}}$ frequency of applied light-dark period during BPV experiments, *NM* not meaningful.

^a^$$f_{max}$$

^b^$$f_{mid}$$.

## Conclusions

This work demonstrated how the Hilbert Huang Transform may be applied to analyse bioelectrochemical signals. Three key phenomena have been proposed that lead to the deviation of IMFs extracted from BPV physical signals, to those extracted from synthetic square waves: (i) time delay between illumination and exoelectrogenesis; (ii) circadian-controlled *psbAI* (PSII D1:1 protein gene) expression rhythm; and (iii) circadian-controlled diurnal intracellular glycogen rhythm. A key limitation of the study is the sensitivity of EMD-based algorithms to the delta/step function. A simple improvement to the study is to repeat the experiments under constant light. This will eliminate the steep changes in current magnitude at the light-dark transitions and also confirm the presence of circadian rhythms in exoelectrogenesis. To test the hypotheses and further interrogate the physical meaning of the modes, the experiments and analysis conducted in this work should be repeated with mutant cells genetically altered to test the proposed hypothesis (e.g., $\Delta$*psbAI *mutants to test the hypothesis for the genesis of mode $\tilde{d}5_{|3\;h}$/$\tilde{d}7_{|12\;h}$, $\Delta$*ldpA *mutants to test the hypothesis for the shortening of period $\tilde{d}7-8_{|12\;h}$ under iron starvation etc.). The experiments and analysis should also be repeated in the presence of site specific respiratory and photosynthetic electron transport chain inhibitors which are expected to affect modes $\tilde{d}1-4$ and $\tilde{d}5-6_{|12\;h}$. Further, due to other limitations in EMD-based algorithms such as sensitivity to noise and sampling, additional techniques should also be tried and the results compared to those presented here. Techniques such as Synchrosqueezed wavelet transforms, Empirical wavelet transform, Variational mode decomposition, and Fourier decomposition method highlighted in the introduction could be tried. Circadian influence in exoelectrogenesis as proposed in this work may offer an explanation for the difference in patterns (amplitudes, timings of peaks and troughs etc.) of exoelectrogenic signals across cyanobacteria species. For example, *Synechocystis* is known to have lower amplitude and less accurate circadian rhythms than *S. elongatus *^43^. This is consistent with higher peaks and troughs reported in *Synechococcus* chronoamperograms^8^. Finally, a confirmation of the hypotheses developed in this work may offer a bioelectrochemical approach for performing simple chronobiology experiments that eliminates the need for engineering reporter strains and extensive fluorescence measurements typical in the study of cyanobacteria circadian rhythms, while increasing the time resolution of experiments.

## Methods

### Culturing and characterisation

All cultures were grown at 30 $^{\circ }C$, a white light intensity of 21.0 ± 0.3 $\upmu$mol m$^{-2}$ s$^{-1}$ and a shaking speed of 120 *rpm*.

#### Stock culture

A stock culture of *Synechococcus elongatus* sp. PCC7942 (Pasteur Culture Collection) was grown in liquid blue-green BG11 media^44^. The media was replenished when the stock culture entered the decline phase. The stock culture was tested for axenicity prior to starting the experimental cultures by plating on tryptic soy plates and leaving in the dark to ensure no growth.

#### Experimental cultures

Experimental cultures were started by re-suspending biomass pellets obtained from the stock culture in exponential phase by rapid centrifugation (4000 $\times$ g for 10 min) in fresh media following two washing steps. The cultures were grown for 4 days (96 *h*), before inoculating in the BPV devices.

For the iron deplete cultures, Fe(−)|Air and Fe(−)|20% CO${_2}$, the BG11 medium was modified by replacing ammonium ferric citrate by an equal molar amount of ammonium citrate^7^. In addition, the medium for the Fe(−)|20% CO${_2}$ cultures was buffered to pH 7.0 using HEPES-NaOH to prevent excessive acidification of the culture by CO${_2}$. The 20% CO${_2}$ atmosphere was induced by growing the cultures in Erlenmeyer flasks with two port caps. The inlet was supplied by a 20% v/v CO${_2}$ gas supply at 10 ml min$^{-1}$. Supplementary Fig. S7 shows the P&ID of the experimental set-up. The inlet and outlet ports were fitted with 0.2 $\upmu$m filters to maintain a sterile environment within the flasks.

All glassware used to prepare media and grow the iron depleted cultures were soaked in nitric acid overnight to reduce traces of solids and therefore iron to a minimum^7^. All media were prepared with Millipore$^{\circledR }$ ultra pure reverse osmosis water.

#### Determination of cell number

Cell number, N (cells ml$^{-1}$), was estimated from optical density at 750 nm (OD750). To account for the effects of cell size and medium composition on optical density readings, independent standard curves for converting OD750 readings to N were calibrated for each growth condition^45^. To generate the curves, OD750 measured using a Thermo Scientific Evolution 201 UV–visible spectrophotometer, and N measured using a Beckman Coulter$^{\hbox {TM}}$ Z2 particle counter, were recorded over 216 hours (Supplementary Fig. S1a–c). A second order polynomial was then fit to the data to produce equations for estimating N from OD750 (Supplementary Fig. S1d–f and Supplementary Table S1).

#### Determination of chlorophyll *a* content and cell size

Chlorophyll *a *content and cell size were determined as described in previous work^25^.

### BPV device and operation

BPVs were operated at 30 $^{\circ }C$ and a white light intensity of 24 ± 0.3 $\upmu$mol m$^{-2}$ s$^{-1}$.

#### BPV architecture

BPV architecture and assembly was as described in recent work^12^. To recap, the membrane electrode assembly (MEA) was made up of a porous Toray carbon paper anode, a nitrocellulose membrane (0.22 $\upmu$m pores), and an Alfa Aesar platinum coated carbon paper cathode with 3 mg m$^{-2}$ Pt loading. The growth medium served as the electrolyte and no exogenous mediators were used.

For the Fe(−)|20% CO${_2}$ condition, a 20% CO${_2}$ atmosphere was induced by sealing the anode chamber with a two port cap, and supplying the inlet with 10 ml min$^{-1}$ of a 20% v/v CO${_2}$ gas supply. The inlet and outlet were fitted with 0.2 $\upmu$m filters to maintain a sterile environment (Supplementary Figs. S7–S8).

#### Operation

BPVs were inoculated with 5 ml of culture started from biomass pellets harvested from the experimental cultures (4000$\times$*g* for 10 min) after four days of growth. The pellets were resuspended in fresh medium to a cell concentration of 6.78 $\times$ 10$^{8}$ cells ml$^{-1}$ after one washing step. BPVs were left to stand at open circuit for four days under a 12 h:12 h light-dark cycle to allow cells to colonise the carbon anodes and form a biofilm (Supplementary Fig. S9a). Following biofilm formation, the operating procedure shown in Table 2 was followed. Voltage measurements were taken every 2 min (120 s) and converted to current density *J* using Ohm’s law,

1 $$\begin{aligned} J=\frac{V}{R\times A}, \end{aligned}$$

where *V* is the measured voltage, *R* the applied external load, and *A* the electrode geometric area.

**Table 2:** Operating procedure for BPVs.

1.	Open circuit potential (OCP) measured
2.	33 M$\Omega$ external resistor connected; light-dark period set to 3 h:3 h
3.	Voltage allowed to stabilise; repeatable photoresponse recorded
4.	Polarisation curve measured in the dark^a^
5.	33 M$\Omega$ external resistor connected; light-dark period set to 12 h:12 h
6.	Voltage allowed to stabilise; repeatable photoresponse recorded
7.	Polarisation curve measured under illumination
8.	Media refreshed; 33 M$\Omega$ external resistor connected; light-dark period set to 3 h:3 h^a^
9.	Voltage allowed to stabilise; repeatable photoresponse recorded^a^

^a^Excluding Fe(−)|Air BPVs.

### Statistical analysis

Cultures were grown in three independent replicates for each growth condition and three independent BPVs were run for each growth condition. All readings were conducted for each replicate and means taken. For confocal image processing, the weighted average means and standard deviations were calculated to account for the different number of cells in the image of each sample. Student’s t tests at 5% significance level was used to test the difference in cell sizes.

### Hilbert–Huang transform

#### Hilbert transform

The Hilbert transform enables the identification of the instantaneous frequency and energy of a signal by creating an analytic signal (a complex-valued function) from a measured time series.

For a time series *X*(*t*), its Hilbert transform *Y*(*t*) is given by Eq. (2),

2 $$\begin{aligned} Y(t)=\frac{1}{\pi }P\int _{-\infty }^{\infty }\frac{X(\tau )}{t-\tau }d\tau , \end{aligned}$$

where P is the Cauchy principal value. The signal *X*(*t*) and its Hilbert transform *Y*(*t*), both real, are then paired to form the analytic signal *Z*(*t*). The analytic signal is a complex number with *X*(*t*) as the real part and *Y*(*t*) the imaginary part. The analytic signal, *Z*(*t*), can then be represented in polar form using Euler’s formula, Eq. (3),

3 $$\begin{aligned} Z(t)=X(t)+iY(t)=a(t)e^{i\theta (t)}, \end{aligned}$$

where *i* is the imaginary unit. The modulus of the complex number, *a*(*t*), is defined as the instantaneous energy and is given by

4 $$\begin{aligned} a(t)=\left[ X^{2}(t)+Y^{2}(t)\right] ^{\frac{1}{2}}, \end{aligned}$$

while the argument of the complex number, $\theta (t)$, is defined as the instantaneous phase of *X*(*t*) and is given by

5 $$\begin{aligned} \theta (t)=\arctan \left( \frac{Y(t)}{X(t)}\right) . \end{aligned}$$

Finally, the instantaneous frequency of *X*(*t*) is defined as derivative of the instantaneous phase, $\omega (t)$,

6 $$\begin{aligned} \omega (t)=\frac{d\theta (t)}{dt}. \end{aligned}$$

Since the Hilbert transform defines instantaneous frequency through differentiation, several points are required to calculate a stable derivative at a point. Hilbert transform data therefore needs to be oversampled in comparison to Fourier analysis^13^. The highest stable frequency that can be extracted by the Hilbert transform is therefore limited to,

7 $$\begin{aligned} f_{max}=\frac{1}{n\Delta t}, \end{aligned}$$

where *n* is the number of sampling intervals ($\Delta t$) between the minimum data points required. In the original implementation of EMD, a minimum of five data points was given as a heuristic^13^. Using this heuristic, $n=4$, and highest stable frequency the can be extracted with the data collected with a sampling interval of 120 s is 2.1 mHz. However, to interpret $\omega (t)$ as the instantaneous frequency, some restrictions apply. First, given that Eq. (6) is a single value function, i.e., there is only one frequency $\omega$ at a given time t, the data to be transformed must also represent only one component at the time t^13^. Physically, this may be conceptualised as the oscillating data having the same number of zero mean crossings and extrema (maxima, minima) per unit time. Secondly, the data must be symmetric around the *local* *zero mean* level, otherwise Eq. (6) may result in physically meaningless negative frequencies^13^. The Empirical Mode Decomposition (EMD) was developed to decompose nonlinear and/or non-stationary signals into components that meet the above restrictions.

#### Empirical mode decomposition (EMD)

The goal of EMD is to decompose a signal that contains multiple dynamic oscillations superimposed on each other, into a finite set of physically meaningful components^13,42^. One key attribute of EMD is the definition of the *local mean* of a signal as the mean of the upper and lower envelopes determined from its local extrema. That is, the separate lines connecting the local maxima [upper envelope] and local minima [lower envelope] by a smooth interpolation over the timespan of the signal.

With the local mean as the reference point for oscillations, high frequency oscillations (oscillations around the local mean) can be extracted from the low frequency oscillations (embedded within the local mean) in an iterative sifting process. The result is a series of components, from high to low frequency, called intrinsic mode functions (IMFs), each encapsulating oscillations of a characteristic timescale (time lapse between successive extrema) intrinsic to the data. For any given IMF, each cycle between two zero crossings contains only one mode of oscillation, i.e., with no superimposed riding waves^13^.

The formal definition of an IMF as given in the original implementation of EMD, is a function that satisfies two conditions^13^:

1. The number of extrema and the number of zero crossings are equal or differ at most by one.
2. The mean value of the envelope defined by the local maxima and the envelope defined by the local minima is zero at all points.

The first condition limits each IMF to one component at any point in time (no riding waves), allowing calculation of instantaneous frequency using Eq. (6). The second condition ensures symmetry around the local mean to prevent negative frequency values. Algorithm 1 shows the EMD pseudo-code.

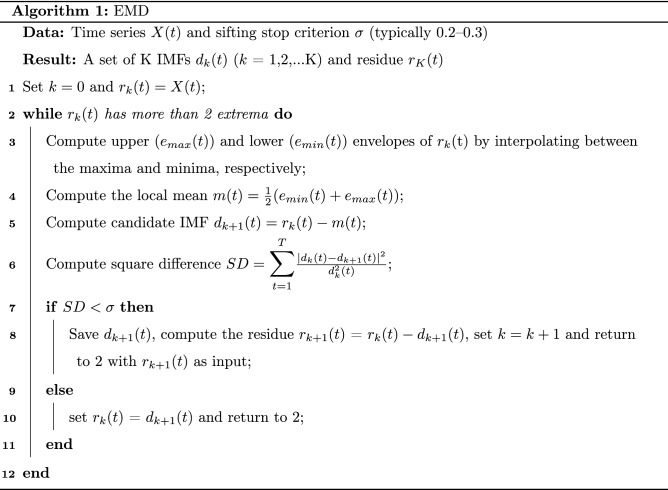



The original signal can be reconstructed as the sum of the IMFs and the final residue which is a monotonic function,

8 $$ X(t) = \sum\limits_{{k = 1}}^{K} d_{k} (t) + r_{K} (t).$$

It should be noted that since the local mean changes over the course of the signal, what is considered a high frequency in one part of the signal may be considered low frequency in another part of the signal. Therefore, both the frequency and the amplitude of the resultant IMFs are allowed to vary with time (AM-FM), adapting to the local characteristics of the signal. A caveat of this adaptability is that one IMF may include oscillations with vastly differing timescales, or oscillations of similar timescales may occur in different IMFs^19^. This is termed ‘mode mixing’. To reduce mode mixing, several improvements to the EMD have been proposed, including the Ensemble EMD (EEMD), Complete EEMD with Adaptive Noise (CEEMDAN), and most recently the Improved CEEMDAN (ICEEMDAN)^19,46,47^. In this work, ICEEMDAN is used to obtain similar timescales in each IMF.

#### Improved complete ensemble EMD with adaptive noise

To reduce mode mixing, the ICEEMDAN approach creates copies of the residue $r_{k}$ at each stage of decomposition and adds the *k*th IMF of different realisations of controlled white Gaussian noise to each copy, resulting in an ensemble of noisy residues^19^. Then, the local mean of the residue is taken to be the average of the local means of its noisy copies. This helps improve the estimation of the local mean, resulting in IMFs of similar scale^19^. In Algorithm 1, the operator $E_{k}(\cdot )$ produces the *k*th IMF via EMD, $M(\cdot )$ produces the local mean of the signal it is applied to (as in step 4 in algorithm 2), $\left\langle \cdot \right\rangle$ is the action of averaging over the different copies of residue plus noise, and $std(\cdot )$ is the standard deviation.

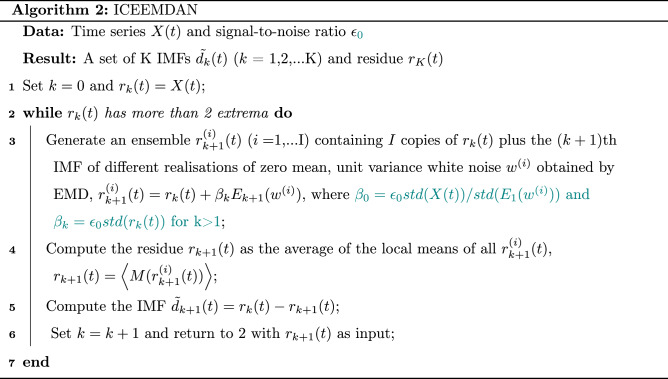



In this work, ICEEMDAN was implemented using the Matlab code developed in the original implementation of the method^19^. The noise standard deviation was set to 0.2 $\le$ *std* $\le$ 0.3 for all decompositions, with values used for individual profiles set to minimise mode mixing (determined visually). In addition, signal-to-noise ratio ($\epsilon _{0}$) was set to increase at every stage to account for the reducing energy of the residuals as described in the original implementation. Other parameters used were set as follows: number of realisations per ensemble (I) = 500; maximum number of sifting iterations = 5000.

#### Hilbert spectrum and marginal spectrum

After ICEEMDAN decomposition of *X*(*t*), the Hilbert transform can be applied independently to each IMF $\tilde{d}_{k}(t)$, so that the analytic signal in Eq. (3) can be re-written as the sum of the analytic signals of the K IMFs,

9 $$ Z(t) = \sum\limits_{{k = 1}}^{K} {a_{k} (t)exp\left( {i\smallint \omega _{k} (t)dt} \right).}$$

In Eq. (9), the final residue $r_{K}(t)$ is intentionally excluded since there is some uncertainty whether the monotonic component is part of a longer oscillation and the instantaneous energy of $r_{K}$ can be overpowering relative to the energies of $\tilde{d}_{k}$^13^.

The information in Eq. (9) can be represented in a three-dimensional diagram called the ‘Hilbert spectrum’, $H(\omega ,t)$, with *t* on the x-axis, $\omega _{k}(t)$ on the y-axis, and $a_{k}(t)$ contoured on the frequency-time plane for all K IMFs^13^.

In addition, the marginal spectrum, $h(\omega )$, is defined as

10 $$\begin{aligned} h(\omega )={\int }_{0}^{T}H(\omega ,t)dt, \end{aligned}$$

and provides a measure of the total contribution from each frequency value to the variable amplitude.

## Supplementary Information

Supplementary Information.
